# Isolation and characteristics of multi-drug resistant *Streptococcus porcinus* from the vaginal secretions of sow with endometritis

**DOI:** 10.1186/s12917-020-02365-9

**Published:** 2020-05-20

**Authors:** Yawen Wang, Haiyong Guo, Yun Bai, Tanqing Li, Ruitao Xu, Tairan Sun, Jicheng Lu, Qinye Song

**Affiliations:** 1 Hebei Province Research Center of Veterinary Biological Engineering and Technology, College of Veterinary Medicine, Hebei Agricultural University, Baoding, 071000 China; 2 School of Life Science, Jilin Normal University, Siping, 136000 China; 3 Baoding Animal Disease Control and Prevention Center, Baoding, 071000 Hebei China

**Keywords:** Sow, Endometritis, *Streptococcus porcinus*, Multi-drug resistance

## Abstract

**Background:**

Sow endometritis is a common disease in pig breeding farms after artificial insemination, which leads to gray-green vaginal secretions and decreased conception rates. It is important to perform an etiologic diagnosis for effective treatments and control of diseases. The aim of this study was to carry out a pathogenic detection in five specimens of vaginal secretions collected from sick pigs with endometritis, implement identification of the pathogens by phenotypic detection and 16 s rDNA sequence and phylogeny analysis, and determinate antibiotic susceptibility of the isolates.

**Results:**

A *Streptococcus* strain was isolated and identified from all of the five specimens. The isolate was positive for Voges-Proskauer (V-P) and for the hydrolysis of arginine, esculin and myelin-associated glycoprotein (MAG). Acid formation was observed for sorbitol, mushroom sugar, sucrose, and glucose. The 16S rDNA sequence of the isolate possessed 99.93% similarity to that of *Streptococcus porcinus*. The phylogenetic analysis of 16S rDNA sequence showed that the isolate belonged to the same clade as the *S. porcinus* strains from humans, pigs, and other animals. The isolate exhibited multi-drug resistance to aminoglycosides, quinolones, macrolides and tetracyclines except being sensitive to some β- lactams such as penicillin G, cephalothin, cefazolin, cephradine and cefuroxime.

**Conclusions:**

A *S. porcinus* isolate with multi-drug resistance was identified from vaginal secretions of sows with endometritis in one pig breeding farm, which suggests that the sow endometritis was caused by *S. porcinus* infection during artificial insemination. This study indicates that sensitive antibiotics such as penicillin G or some cephalosporins could be used for treatment of the diseases. In addition, the study hints that bacterial multi-drug resistance is a tough problem for disease treatment in pig farms.

## Background

Porcine endometritis is a common genital disease in sows, which is induced by the infection by bacteria, viruses, parasites, fungal toxins, and other pathogenic factors [1–3]. The pigs with endometritis often exhibit a decreased appetite, fever, extrados, brown mucous or purulent discharge from the vagina, anestrus or irregular estrus, infertility or miscarriage breeding. Moreover, the porcine endometritis can cause severe sepsis and even death [4, 5]. *E. coli, Streptococcus, Staphylococcus, Corynebacterium, Pseudomonas aeruginosa,* and *Proteus* are major pathogens that cause endometritis [1, 6]. In some large-scale farms of China, 47.6% of the sows are culled for various anomalies among which 41.4 and 17.7% are eliminated due to endometritis and other reproduction-related diseases, respectively, 18.5% result from limb hoof disease, and the remaining part is attributed to other pathologic conditions [4].

The species *Streptococcus porcinus* belonging to the genus *Streptococcus* is commonly associated with pyogenic infections, abortion and endocarditis of pigs, as well as genitourinary tract infection of women [7–9]. *S. porcinus* was first identified in pigs in 1984, and then isolated from bovine milk [7, 8]. The organism has also been detected in the urogenital tracts of pregnant women [9–11]. *S. porcinus* is usually detected in pig tonsils. 19.5% of tonsils of the pigs in slaughterhouses were positive for the isolation of *S. porcinus*, which is just less than the prevalence of *S. suis* infections (53.7%) [12]. Swine *S. porcinus* causes pig lymph node abscess, throat abscess, pneumonia, arthritis, and endocarditis, as well as sow abortion [1, 8, 13, 14], but there are rare reports on sow endometritis caused by *S. porcinus*.

In this study, to confirm the cause of a sow endometritis case from a pig breeding farm in Hebei Province, China, five vaginal secretion samples from the sick sows were detected in etiology by isolation and culture, phenotypic examinations and 16S rRNA sequence analysis of pathogen. In addition, antibiotic susceptibility assays of the isolate were carried out in order to instruct an effective treatment of the disease.

## Results

### Cultural and morphological characteristics of the isolate

**Fig. 1:**
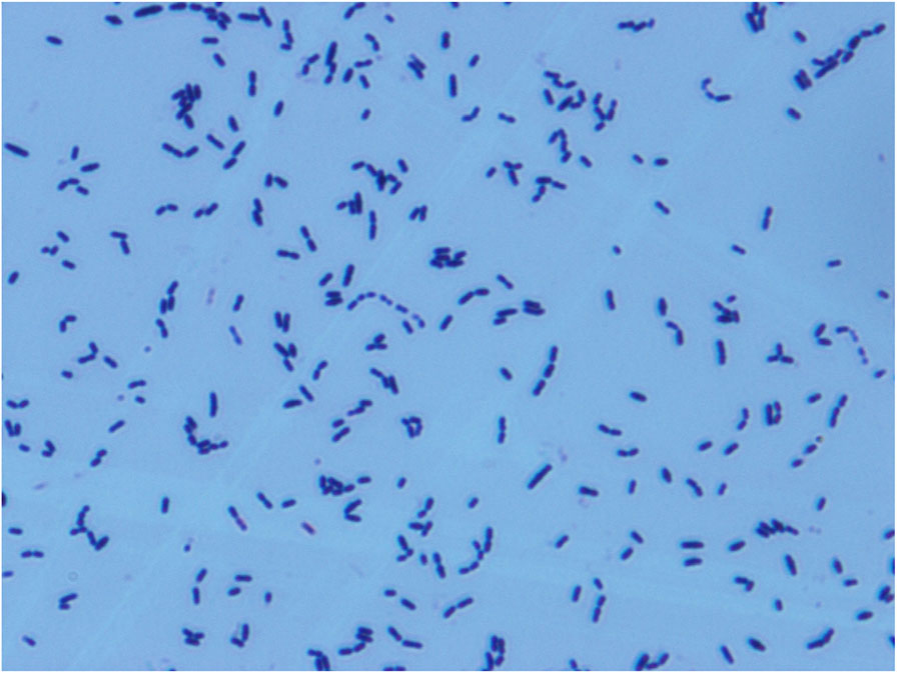
Morphological characteristics of the isolate in solid medium under the microscope (×1000)

Bacteria with similar cultural and morphological characteristics were isolated in all vaginal secretion samples from five sick pigs. The bacterial isolates showed a poor growth on ordinary nutrient agar with fewer small tip-sized colonies, but a good growth on blood agar and serum agar plates with off-white, smooth-surfaced, and regular-edged microcolonies. And transparent β-type hemolysis was present around the colonies on the blood agar plate. The isolates did not grow on the eosin methylene blue and MacConkey agar plates. The bacteria precipitated at the bottom of the tube containing serum broth after overnight incubation. The microscopy analysis revealed that the isolates appeared gram-positive cocci in a single, pairs, or short-chain arrangement (Fig. 1). No similar cultural and morphological bacteria were detected from the vaginal secretion samples of all healthy pigs.

### Physiological characteristics of the isolate

To differentiate whether the isolated bacteria was *Streptococcus* or *Staphylococcus*, we performed a catalase test. The bubbles observed for positive controls after dropping 3% hydrogen peroxide solution onto the staphylococcal reference strain. In contrast, no bubbles generated for the isolated bacteria and the negative control after adding 3% hydrogen peroxide onto the isolated bacteria and the streptococcal reference strain, which indicated that the isolated strains were catalase-negative. Further biochemical assays revealed that the isolates were positive for Voges-Proskauer (V-P) and for the hydrolysis of arginine, esculin and myelin-associated glycoprotein (MAG). Negative results were observed for the production of dipeptidyl aminopeptidase (DPP), polymethylgalacturonase (PMG) and for hydrolysis of temozolomide (TMZ). Acid formation exhibited for sorbitol, mushroom sugar, sucrose, and glucose, but did not for lactose, raffinose, xylose, and adonitol (Table 1).

**Table 1:** Phenotypic characteristics of the isolated bacteria

Phenotypic characteristic	Reaction	*S. porcinus*^a^
Catalase	-^b^	–
Voges-Proskauer (V-P)	+ ^c^	+
Arginine	+	+
Esculin	+	+
Polymethylgalacturonase (PMG)	–	–
Temozolomide (TMZ)	–	–
Dipeptidyl aminopeptidas (DPP)	–	–
Myelin-associated glycoprotein (MAG)	+	+
Sorbitol	+	+
Adonitol	–	/ ^d^
Mushroom sugar	+	+
Lactose	–	–
Xylose	–	/
Sucrose	+	+
Glucose	+	/
Raffinose	–	–

^a^Results indicated by the manufacturer for the identification of *S. porcinus* and the references (Martin et al., [9]; Duarte et al., [10])

^b^Negative reaction; ^c^ Positive reaction; ^d^ no data

### Phylogenetic analysis of 16S rRNA gene sequence of the isolate

In order to genetically classify the isolated streptococci, we amplified the 16S rDNA sequence. PCR products of expected size were obtained after PCR using bacterial 16S rRNA gene sequence-specific primers. DNA sequencing analysis showed that the size of the amplified nucleic acid sequence was 1428 bp (GenBank accession number: MN148888).

Pairwise alignment of the isolated strain with reference streptococcal strains revealed that the 16S rRNA gene sequence of the isolated strain had about 99.93, 98.18, 96.60, 96.64, 96.78 and 96.33% similarities to those of *S*. *porcinus*, *S. pseudoporcinus*, *S. uberis*, *S. parauberis*, *S. canis* and *S. dysgalactiae*, respectively, from human or animal.

**Fig. 2:**
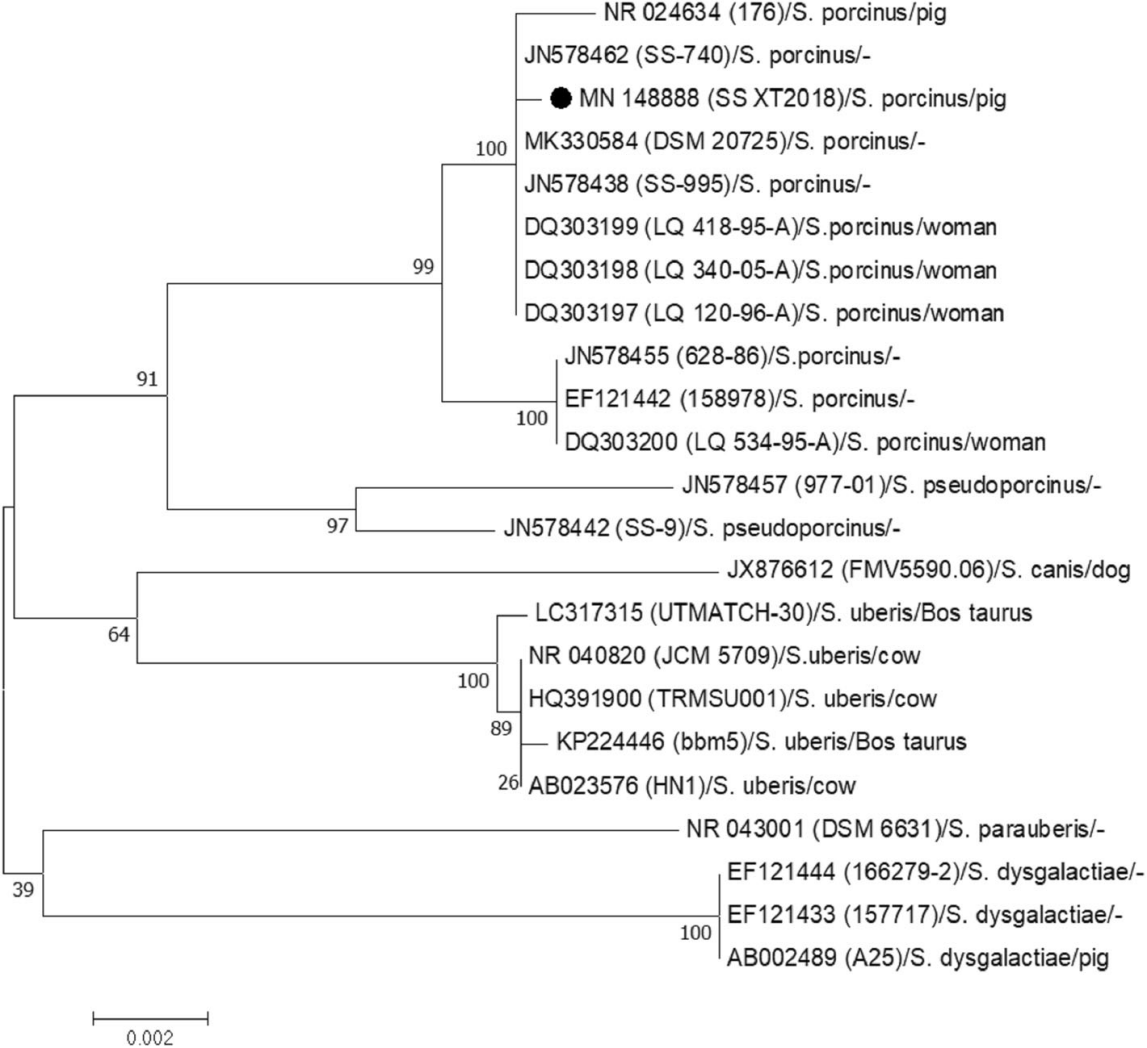
Phylogenetic tree of the isolated strain based on 16S rDNA sequence. ● The isolate

The phylogenetic tree based on 16S rDNA sequences suggested that the isolated strain was in the same small branch with the human or animal *S. porcinus* strains in GenBank, which indicated a closer genetic relationship between the isolated strain and *S. porcinus* strains compared to other subspecies. The isolate was in different small branches with *S. pseudoporcinus*, but co-located in a large branch, indicating a closer genetic relationship with *S. pseudoporcinus*. However, it situated in distinct branches with *S. uberis, S. canis, S. dysgalactiae*, and *S. parauberis* strains, which displayed a distant genetic relationship (Fig. 2).

### Multi-drug resistance of the isolate

In order to clarify further biological characteristics of the isolate, we conducted antibiotics susceptibility assays. The results were shown in Table 2. Among tested 25 different antibiotics, the isolate was only sensitive to penicillin G, cephalothin, cefazolin, cephradine, and cefuroxime, but it was resistant to most antibiotics such as lincomycin, nitrofurantoin, rifampicin, aminoglycosides, quinolones, macrolides, and tetracyclines. The results demonstrated that the isolate is multi-drug resistant.

**Table 2:** Drug susceptibility of the isolated bacteria

Antibiotics	Drug contents (μg/piece)	Criteria for inhibition zone (mm)			Measured inhibition zone (mm)	Results
		Resistance (R)^a^	Intermediate (I)^b^	Sensitivity (S)^c^		
β- lactams						
Ampicillin	10	≤18	19–25	≥26	19.87 ± 0.23	I
Penicillin G	10 IU	≤19	20–27	≥28	28.10 ± 0.17	S
Amoxicillin	20	≤13	14–17	≥18	0	R
Cephalothin	30	≤14	15–17	≥18	25.03 ± 0.06	S
Cefazolin	30	≤14	15–17	≥18	24.00 ± 0.10	S
Cephradine	30	≤14	15–17	≥18	29.10 ± 0.17	S
Cefuroxime	30	≤14	15–22	≥23	27.00	S
Cefotaxime	30	≤25	26–27	≥28	0	R
Ceftriaxone	30	≤25	26–27	≥28	26.07 ± 0.12	I
Aminoglycosides						
Amikacin	30	≤14	15–16	≥17	0	R
Streptomycin	10	≤11	12–14	≥15	0	R
Kanamycin	30	≤13	14–17	≥18	0	R
Macrolides						
Erythromycin	15	≤13	14–22	≥23	0	R
Roxithromycin	15	≤13	14–22	≥23	0	R
Acetylspramycin	30	≤13	14–22	≥23	0	R
Tetracyclines						
Tetracycline	30	≤18	19–22	≥23	2.93 ± 0.06	R
Minocycline	30	≤14	15–18	≥19	8.90 ± 0.17	R
Doxycycline	30	≤12	13–15	≥16	2.00	R
Quinolones						
Ofloxacin	5	≤12	13–15	≥16	0	R
Ciprofloxacin	5	≤15	16–20	≥21	0	R
Norfloxacin	10	≤12	13–16	≥17	0	R
Enrofloxacin	10	≤12	13–16	≥17	0	R
Lincomycin	2	≤14	15–20	≥21	10.97 ± 0.06	R
Macrodantin	300	≤14	15–16	≥17	0	R
Rifampicin	5	≤16	17–18	≥19	17.67 ± 0.58	I

^a^Resistance; ^b^ Intermediate; ^c^ Sensitivity

## Discussion

Endometritis is one of the major diseases causing a high sow elimination rate and 6.6% of breeding sows death in large-scale farms [15]. Many pathogens, including virus such as Japanese encephalitis, porcine reproductive and respiratory syndrome virus, and pseudorabies virus, bacteria such as *E. coli*, *Streptococcus*, *Staphylococcus*, *clostridia*, *Pseudomonas aeruginosa,* and parasites (e.g. *trichomonas*), can cause pig endometritis [1–6]. If disinfection is not strict, the breeding process, including artificial insemination, birth, midwifery, and abortion, can lead to infections and result in endometritis [1, 16, 17].

To improve the breeding and reproduction rate, artificial insemination is widely used in intensive pig farms. However, semen is easily contaminated with microbial pathogens carried by boar or contaminated during semen fluids collection and storage process [16, 17]. In the current study, the clinical endometritis cases were likely correlated with contamination of semen and artificial insemination because symptoms of endometritis emerged just 3 to 5 days after insemination. Therefore, we carried out an etiological examination to determine its causes.

In order to ensure the accuracy of detection results, we combined phenotypic and molecular biological approaches to determine the causative agent of sow endometritis, and set up vaginal secretion samples of clinical healthy sows and gilts in the same farm as the case controls. In addition to bacteriological testing, PCR was employed for detecting classical swine fever virus (CSFV), porcine reproductive and respiratory syndrome virus (PRRSV), porcine circovirus type 2 (PCV2) and porcine circovirus type 3 (PCV3) in the sow vaginal secretions and did not find these viral nucleic acids (supplementary data). These results suggest that the sow endometritis was caused by a bacterial infection most likely due to contamination of semen and artificial insemination.

Indeed, we isolated and identified a Gram-positive *Streptococcus* from the vaginal secretions of the sick sows based on Gram-staining, microscopy, catalase and biochemical assays. The isolated *Streptococcus* with β-hemolysis in short chains arrangement under the microscope is negative to catalase, and positive to V-P test and to acid formation of sorbitol, mushroom sugar, sucrose and glucose, and hydrolysis of arginine and esculin. The most of these characteristics are consistent with those of *Streptococcus porcinus* in spite of exhibition of some variable reaction in the study [9, 10].

16S rRNA gene sequences contain highly and moderately conserved sequence regions, and highly variable sequence region, which is a powerful molecular target and widely used in classification and identification of bacteria from clinical isolates [18–20]. 16S rRNA gene sequencing is a very useful method for differentiating *S. porcinus* from *S. pseudoporcinus* isolates, and for determining the taxonomic status of isolates from animal, human and dairy sources [21, 22]. In order to further clarify the subspecies of the *Streptococcu*s isolate in this study, we amplified the16S rRNA DNA sequence and conducted a comparative phylogenetic analysis. The results suggest that the *Streptococcus* isolate from vaginal secretions is likely to be *Streptococcus porcinus* because its 16S rRNA sequence is highly similar to those of the *Streptococcus porcinus* reference strains from both humans and pigs.

Bacterial resistance is an important global concern of public health especially in the last 20 years. The rapid increase in drug-resistant strains has caused a serious problem in clinical treatments of human and animal infectious diseases [23–25]. Resistant isolates of animal origin are very common and easily disseminated between animal and humans from contaminated food [24]. Wang et al. [25] reported that multidrug-resistant *Salmonella Typhimurium* isolates dramatically increased among the *salmonella* isolated from human, animal and retail milk between 2011 to 2016. Resistances of *S. porcinus* for erythromycin, minocycline, sulfamethoxazole-trimethoprim, streptomycin and tetracycline were described in previous study [26]. In this study, we observed that the *S. porcinus* strain had multi-drug resistance to antibiotics including aminoglycosides, quinolones, macrolides, and tetracyclines except being sensitive to some β- lactams such as penicillin G, cephalothin, cefazolin, cephradine and cefuroxime. These data suggest that sensitive antibiotics such as penicillin G or some cephalosporins could be used for treating this disease through local delivery into the uterus following washing and sterilizing vulva with disinfectant fluid. Meanwhile, it is necessary to implement more strict clinical veterinary medicine practice, including disinfection procedure during semen collection and artificial insemination.

Though an experimental proof would be of high interest for demonstrating the occurrence of endometritis during artificial insemination by detection of semen and instruments for insemination or animal experiment, we did not do these tests in this study because the semen and other relevant insemination materials had not been gotten then, and animal experiment was limited in fields. Nevertheless, we estimated that the occurrence of endometritis could be associated with artificial insemination since sows unfertilized artificially were not attacked by this disease in this farm. In addition, it happened just on days 3–5 after artificial insemination.

In summary, the identification of *S. porcinus* provides important information for determining the cause of sow endometritis. The evaluation of antibiotic susceptibility of the isolate could contribute to the treatment of sow endometritis from bacterial infection. It should be noted that bacterial multi-drug resistance is a great challenge to the current disease control.

## Conclusions

This study demonstrates that the sow endometritis was caused by *S. porcinus* infection during artificial insemination in the pig breeding farm. The isolate has multi-drug resistance to most antibiotics applied clinically, which may be a great challenge for the control of animal infectious diseases. In addition, sensitive antibiotics such as penicillin G or some cephalosporins could be used for treating sow endometritis by *S. porcinus* infection in pig farm.

## Methods

### Case presentation

Eighteen multiparous sows and twenty-four replacement gilts had a fever and lost appetite 3 to 5 days after artificial insemination and generated large amounts of pale yellow vaginal secretions. On the second estrus, endometritis occurred, and gilts estrus conception rate was significantly decreased or breeding capacity was totally abolished. Five random sick pigs’ vaginal secretions (3 from sows and 2 from gilts) were collected aseptically using 10 mL disposable syringes with needles removed and put into 15 mL sterile collection tubes for laboratory testing on day four after presentation of the clinical signs. Five of vaginal secretion samples were subsequently collected from three healthy sows and two healthy gilts, which were not fertilized by artificial insemination as the case controls. After the samples had been collected, the pigs were released but the diseased ones were separated from the healthy and got clinical treatment.

### Characteristics of bacterial culture

All of the vaginal secretion samples were taken and streaked onto ordinary nutrient agar (Beijing Biotech AOB Star Co., China) and serum-agar plates with 5% sheep serum (Gibco) in ordinary nutrient agar medium. After incubation18 ~ 24 h at 37 °C, colony morphology was examined, Gram stained, and the morphology of bacteria staining was observed under a microscope.

The single colony was re-streaked onto the ordinary nutrient agar, sheep serum agar, sheep blood agar, MacConkey agar, and eosin methylene blue agar plates (Beijing Biotech AOB Star Co., China), respectively, and incubated overnight at 37 °C. Phenotypic characteristics of colonies were further observed. Single colony was picked, inoculated into serum nutrient broth containing 5% sheep serum in the ordinary nutrient broth, and incubated for 16–18 h at 37 °C with shaking at 200 rpm. The bacterial cultures were taken for Gram staining and observed under the microscope.

### Catalase test

To conduct catalase examination, the isolated bacterial colonies were picked and placed on clean glass slides, and the strain ATCC25923 of *Staphylococcus* and the strain ATCC 27335 of *Streptococcus* was as the positive or the negative control, respectively; 3% hydrogen peroxide (Sigma) was dropped onto the bacteria on the slides. The appearance of large bubbles indicated that the catalase was positive, whereas no bubbles indicated that it was negative.

### Biochemical examinations

To perform biochemical assays, the bacterial cells from cultures were inoculated into biochemical identification microtubes (Hangzhou Binhe Microorganism Regent Co., Ltd., China), respectively, and cultured for 24 h at 37 °C according to the manufacturer’s instructions.

### PCR of 16S rRNA gene sequences

The bacterial 16S rRNA gene (rDNA) sequence universal primers 16SF (5′-AGAGTTTGATCCTGGCTCAG-3′) and 16SR (5′-GGTTACCTTGTTACGACTT-3′) were utilized for amplification of 16S rRNA sequence from the isolated bacterial cells. PCR reaction system was set as follows: 25 μL of 2 × Taq Plus MasterMix (Beijing ComWin Biotech Co., Ltd. China), 1 μL upstream and downstream primers, respectively, 2 μL of bacterial suspension cells, and 21 μL ddH_2_O. The reaction program was: pre-denaturation at 94 °C for 3 min, followed by 35 cycles of denaturation at 94 °C for 45 s, annealing at 52 °C for 1 min, extension at 72 °C for 1 min, and a final extension at 72 °C for 10 min. PCR products were detected on 1% agarose gel electrophoresis, purified, and then ligated into the pM18-T vector (TaKaRa Dalian, China). The PCR products were sequenced at Sangon Biotech (Shanghai) Co., Ltd., China.

### 16S rRNA gene sequence analysis

The 16S rDNA sequence from the isolate, designated as SS XT2018 (GenBank ID: MN148888), was compared with the reference sequences in GenBank (Table 3) using CLUSTAL W program of molecular evolutionary genetics analysis software (MEGA), version 7.0. An unrooted phylogenetic tree was constructed using the Neighbor-joining method with the maximum likelihood method and bootstrap tests of 1000 replicates.

**Table 3:** Information of reference *streptococcus* strains

Strains	GenBank accession number	Host	Organism
DSM 20725	MK330584	–	*S. porcinus*
SS-740	JN578462	–	*S. porcinus*
SS-995	JN578438	–	*S. porcinus*
LQ 120–96-A	DQ303197	woman	*S. porcinus*
LQ 340–05-A	DQ303198	woman	*S.porcinus*
176	NR_024634	pig	*S. porcinus*
LQ 418–95-A	DQ303199	woman	*S.porcinus*
628–86	JN578455	–	*S.porcinus*
158,978	EF121442	–	*S. porcinus*
LQ 534–95-A	DQ303200	woman	*S. porcinus*
SS-9	JN578442	–	*S. pseudoporcinus*
977–01	JN578457	–	*S. pseudoporcinus*
JCM 5709	NR_040820	cow	*S.uberis*
HN1	AB023576	cow	*S. uberis*
TRMSU001	HQ391900	cow	*S. uberis*
UTMATCH-30	LC317315	*Bos taurus*	*S. uberis*
bbm5	KP224446	Bos taurus	*S. uberis*
157,717	EF121433	–	*S. dysgalactiae*
A25	AB002489	pig	*S. dysgalactiae*
166,279–2	EF121444	–	*S. dysgalactiae*
DSM 6631	NR_043001	–	*S. parauberis*
FMV5590.06	JX876612	dog	*S. canis*

Note: —, no data

### Antibiotics susceptibility test

K-B disk diffusion method was used for testing the susceptibility of the isolate to antibiotics. Single colony was inoculated into serum Mueller-Hinton (MH) broth (BD/Difco) with 5% sheep serum until a turbidity of culture reached 0.5 McFarland units (equivalent to 1.5 × 10^8^ colony forming unit/mL) at 37 °C. The bacterial broth was spread onto the surface of serum MH agar plates and antibiotics such as β-lactams, aminoglycosides, macrolides, tetracyclines, quinolones, lincomycin, macrodantin and rifampicin (Hangzhou Binho Microbial Agent Co., China) (Table 3) were pasted onto the plates. The inhibition zone diameters on the agar plates were measured 18 to 24 h after incubation at 37 °C. The isolate′s susceptibility to these antibiotics was determined according to the manufacturers’ designated standard. The experiment was carried out three times, and the final inhibition zone diameters were expressed as the mean of repeats.

## Supplementary information

**Additional file 1.** Primers used for RT-PCR detection in this study.

**Additional file 2.** Viral DNA/RNA extraction and PCR.

## Data Availability

The datasets generated and/or analysed during the current study are available in the GenBank repository, https://www.ncbi.nlm.nih.gov/nuccore/MN148888.

## Abbreviations

V-P: Voges-Proskauer
MAG: Myelin-associated glycoprotein
DPP: Dipeptidyl aminopeptidase
PMG: Polymethylgalacturonase
TMZ: Temozolomide
PCR: Polymerase chain reaction
CSFV: Classical swine fever virus
PRRSV: Porcine reproductive and respiratory syndrome virus
PCV2: Porcine circovirus type 2
PCV3: Porcine circovirus type 3
