# Supplementary material for: Isolation and characteristics of multi-drug resistant Streptococcus porcinus from the vaginal secretions of sow with endometritis
Source: BMC Vet Res. 2020 May 20;16:146. doi: 10.1186/s12917-020-02365-9 (PMC7238638; doi:10.1186/s12917-020-02365-9)
Supplement: Supplementary file 1 — Additional file 1. Primers used for RT-PCR detection in this study. [file 12917_2020_2365_MOESM1_ESM.docx]

Table 1 Primers used for RT-PCR detection in this study

| Primer name | Pimer sequence(5′→3′) | Target gene | GenBank accession no. | Annealing temperature (℃) | Prouductsize (bp) |
| --- | --- | --- | --- | --- | --- |
| CSFV-F  CSFV-R | GGACCGTTAAGGCCATTTGC  TATAACACCCGTCCACCCTA | E2 | AF092448 | 55 | 287 |
| PRRSV-F  PRRSV-R | ATGGGCGACAATGTCCCTAA  CTTGATGACACAGGTACAGG | Nsp2 | EU008760 | 57 | 464 (PRRSV)  374 (HP-PRRSV) |
| PCV2-F  PCV2-R | ACGGATATTGTAGTCCTGGT  CAAGGCTACCACAGTCAGAA | ORF2 | AF381175 | 57 | 472 |
| PCV3-F | ACATGCGAGGGCGTTTAC | ORF2 | KT869077 | 56 | 307 |
| PCV3-R | CACTTCTGGCGGGAACTA |  |  |  |  |
