# Supplementary material for: Isolation and characteristics of multi-drug resistant Streptococcus porcinus from the vaginal secretions of sow with endometritis
Source: BMC Vet Res. 2020 May 20;16:146. doi: 10.1186/s12917-020-02365-9 (PMC7238638; doi:10.1186/s12917-020-02365-9)
Supplement: Supplementary file 2 — Additional file 2. Viral DNA/RNA extraction and PCR. [file 12917_2020_2365_MOESM2_ESM.docx]

**Viral DNA/RNA extraction and PCR**

Vaginal secretion samples were centrifuged at 4000 ×g for 10 min at 4°C and to eliminate debris. Viral DNA/RNA was extracted from 200 μL of each suspension using TIANamp Virus DNA/RNA Kit (DP315, Tiangen Biotech, Beijing, China) according to the manufacturer’s instructions, and cDNA was synthesized using M-MLV reverse transcriptase (Promega, USA) for the viral nucleic acids of classical swine fever virus (CSFV), porcine reproductive and respiratory syndrome virus (PRRSV). A PCR reaction volume of 20 μL contained 10 μL 2×Taq Master Mix (Cwbio, China), 0.5 μL of each primer (25 pmol/μL) for CSFV, PRRSV, porcine circovirus type 2 (PCV2) or PCV (3) (Table 1), 2 μL of DNA/cDNA and 7 μL of distilled water. The amplification of the target genes was performed as follows: pre-denaturation at 94 ºC for 3 min, followed by 35 cycles of denaturation at 94 ºC for 30 s, annealing at 55 - 57 ºC for 30 s, extension at 72 °C for 30 s, and a final extension at 72 °C for 10 min. The PCR products were checked by electrophoresis on 1.2% agarose gel (Biomiga, Santiago, USA).
